# A cycle generative adversarial network for improving the quality of four-dimensional cone-beam computed tomography images

**DOI:** 10.1186/s13014-022-02042-1

**Published:** 2022-04-07

**Authors:** Keisuke Usui, Koichi Ogawa, Masami Goto, Yasuaki Sakano, Shinsuke Kyougoku, Hiroyuki Daida

**Affiliations:** 1 Department of Radiological Technology, Faculty of Health Science, Juntendo University, 2-1-1, Hongo, Bunkyo-ku, Tokyo, 113-8421 Japan; 2 Department of Applied Informatics, Faculty of Science and Engineering, Hosei University, 3-7-3, Kajino, Koganei, Tokyo, 184-8584 Japan

**Keywords:** CycleGAN, 4D-CBCT, ART, Image quality correction, Lung cancer

## Abstract

**Background:**

Four-dimensional cone-beam computed tomography (4D-CBCT) can visualize moving tumors, thus adaptive radiation therapy (ART) could be improved if 4D-CBCT were used. However, 4D-CBCT images suffer from severe imaging artifacts. The aim of this study is to investigate the use of synthetic 4D-CBCT (sCT) images created by a cycle generative adversarial network (CycleGAN) for ART for lung cancer.

**Methods:**

Unpaired thoracic 4D-CBCT images and four-dimensional multislice computed tomography (4D-MSCT) images of 20 lung-cancer patients were used for training. High-quality sCT lung images generated by the CycleGAN model were tested on another 10 cases. The mean and mean absolute errors were calculated to assess changes in the computed tomography number. The structural similarity index measure (SSIM) and peak signal-to-noise ratio (PSNR) were used to compare the sCT and original 4D-CBCT images. Moreover, a volumetric modulation arc therapy plan with a dose of 48 Gy in four fractions was recalculated using the sCT images and compared with ideal dose distributions observed in 4D-MSCT images.

**Results:**

The generated sCT images had fewer artifacts, and lung tumor regions were clearly observed in the sCT images. The mean and mean absolute errors were near 0 Hounsfield units in all organ regions. The SSIM and PSNR results were significantly improved in the sCT images by approximately 51% and 18%, respectively. Moreover, the results of gamma analysis were significantly improved; the pass rate reached over 90% in the doses recalculated using the sCT images. Moreover, each organ dose index of the sCT images agreed well with those of the 4D-MSCT images and were within approximately 5%.

**Conclusions:**

The proposed CycleGAN enhances the quality of 4D-CBCT images, making them comparable to 4D-MSCT images. Thus, clinical implementation of sCT-based ART for lung cancer is feasible.

## Background

Stereotactic body radiotherapy (SBRT) has been established as a standard treatment for inoperable early-stage non-small lung cell cancer and oligometastases [1]. The irradiation position in SBRT must be accurate, and high precision image guidance can be accomplished using cone-beam computed tomography (CBCT) attached to a linear accelerator [2, 3]. Specifically, four-dimensional CBCT (4D-CBCT) can visualize tumor movement as a series of computed tomography (CT) images that can be used to locate lung tumors in SBRT [4]. Clinical studies of the use of CBCT in adaptive radiation therapy (ART) have begun, and improvements in the quality of radiation therapy by modifying initial treatment plans with morphological changes during fractionated treatment courses [5, 6] has shown clinical benefits [7, 8]. Conversely, conventional linear accelerator-based ART is a major challenge because of the poor quality of CBCT images, which are affected by X-ray scattering, image lag, beam hardening, and patient movements during scanning [9]. Moreover, 4D-CBCT is reconstructed using cone-beam projection subgroups in different respiratory phases. Therefore, sparse projections in each phase bin cause severe artifacts, deteriorating the Hounsfield unit (HU) values and preventing the creation of accurate ART plans using 4D-CBCT images [10, 11]. An effective method for the reconstruction of sparse projections is the total variation (TV) regularization method, which has been used as a regularization term to smooth out noise and streak artifacts [12, 13]. However, because these approaches use a globally uniform TV penalization, small anatomical structures are inevitably over-smoothed and edge regions degrade. Chen et al. developed a prior contour-based TV method to derive an edge map from high-quality prior planning CT, which enhances edges using images registered to the CBCT [14]. However, this method depends on the accuracy of the image registration between the CT and CBCT images. Therefore, it is necessary for 4D-CBCT to restore the correct HU value.

Recently, with the overwhelming attention to deep learning in the medical imaging field, many deep-learning approaches have been proposed for image related tasks ranging from segmentation and classification to super-resolution and image restoration [15–18]. Jiang et al. [15] improved over-smoothed edge regions in under-sampled CBCT images with TV regularized by a convolutional neural network-based method. Additionally, Landry et al. [18] compared U-Nets trained on three types of corrected CBCT image datasets to improve the image quality of original CBCT images. These studies established a deep-learning architecture that uses paired supervised data, and a small difference in the training images can cause error in the conversion process. A 4D-CBCT image cannot be combined with supervised data matched at pixel level, because respiratory movements mean that the conditions during the 4D-CBCT cannot be exactly reproduced for the four-dimensional multislice CT (4D-MSCT) images. Therefore, accurate image correction is quite difficult to obtain using supervised learning with 4D-CBCT images. Improvements in image quality are needed to perform 4D-CBCT image-based ART, which will enable 4D dose distribution and lead to a more accurate evaluation of therapeutic doses in SBRT for lung cancer.

In this study, we create synthetic 4D-CBCT (sCT) images using a cycle generative adversarial network (CycleGAN) framework and aimed to their use for the possibility of using sCT images in ART planning in SBRT for lung cancer. The CycleGAN model enforces an inverse transformation and achieves highly accurate consistency when the underlying structures are similar, even for mappings in nonlinear domains [19–21]. The CycleGAN method is expected to optimize the quality of 4D-CBCT images, and no previous study has reported the possibility of using these images for ART planning by addressing the sparseness of projection data in 4D-CBCT images. We investigated two controlled experiments: one was correction effect of image noise and projection sparseness, and second was effects of deformation in the 4D-CBCT image with the respiratory motion using the CycleGAN method. To secure a number of training data targeting 2D images, we created the sCT images using the 2D Cycle-GAN and investigated the quality of these images. Restoration of the CT number was investigated using the mean error (ME) and mean absolute error (MAE) in each organ region, and image quality and similarity were evaluated using the peak signal-to-noise ratio (PSNR) and structural similarity index (SSIM). Moreover, the dosimetric accuracy of the sCT image-based dose distribution was investigated to determine if it was comparable in quality to that of the MSCT image-based dose distribution.

## Methods

### Image data acquisition

Thoracic 4D CT images acquired with a CBCT and MSCT were used for training the deep-learning model. These CT images were obtained from a publicly available dataset, the Cancer Imaging Archive, which is an open-access information source created by the US National Cancer Institute [22]. In this dataset, the 4D-CBCT images were acquired using an onboard imager equipped with a kilovoltage X-ray source and flat panel detector (Varian Medical Systems, Palo Alto, CA, USA). In addition, the 4D-MSCT image were acquired using a multi-detector CT using helical scanning. The tube voltage was 120 kV, the thickness of each CT image was 3 mm, the matrix size was 512 × 512 pixels, and the field of view was 50 × 50 cm in the 4D-MSCT images and 45 × 45 cm in the 4D-CBCT images. Because these 4D images were composed of 10 phases divided by one respiratory motion, only the first-phase image (0% phase) was used in each 4D image for training. The 4D-CBCT image was centered on the lung cancer region and included whole lung area; that is, the upper, middle, and lower lung areas. Moreover, the 4D-CBCT image was acquired during the course of radiation treatment, and the 4D-MSCT was acquired at the treatment planning before radiation therapy.

### Image synthesis based on the CycleGAN

The training dataset consisted of 50 slice images per patient for 20 patients, giving a total of 1000 4D-CBCT and 1000 4D-MSCT images. Using this dataset, the sCT images based on the 4D-CBCT images were generated using the 2D CycleGAN. Image pairs were adjusted to the same resolution, 45 × 45 cm in 512 × 512 pixels, using bilinear interpolation, and rigid registration was used on the MSCT image to pair it to the corresponding CBCT image.

**Fig. 1:**
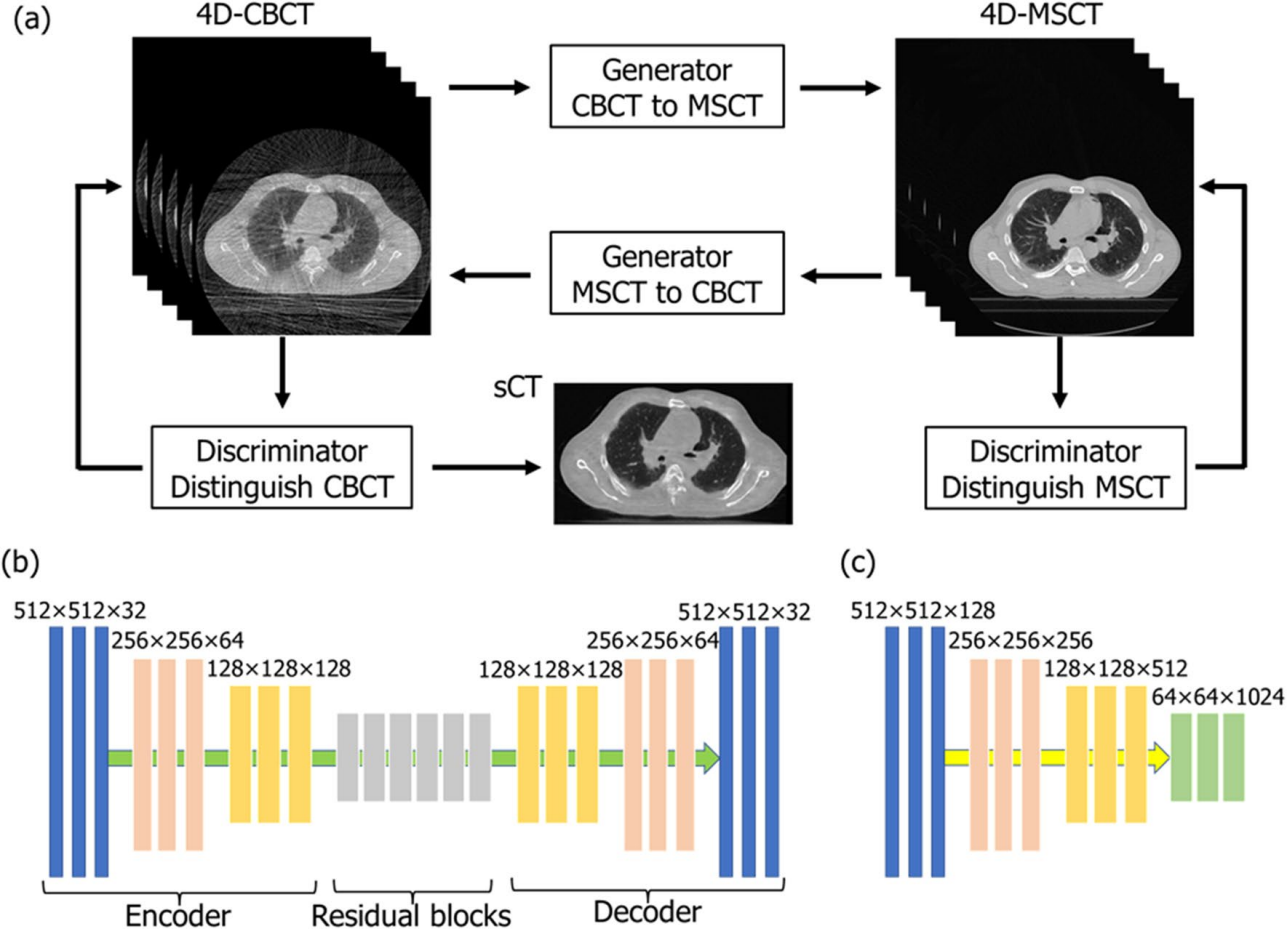
Cycle generative adversarial network (CycleGAN) framework (**a**), and network structure of the generator (**b**) and discriminator (**c**). The training model consists of two generators and two discriminators. To train the CycleGAN, the overall network’s performance is enhanced through networks acting bidirectionally with each other. The sCT image is generated by a network that maps images from a source domain (4D-CBCT) to the target domain (4D-MSCT)

The CycleGAN model consists of four convolutional neural networks and relies on two subnetworks, one generator and one discriminator, which have opposing functions. To train the CycleGAN, all four networks were trained simultaneously to maximize the performance. Because these networks are pitted against each other, each improves its ability, resulting in accurate 4D-CBCT image generation [19, 23]. Figure 1 presents a flowchart of the CycleGAN model, and Table 1 lists the structural details of the generator, discriminator, and gradient optimization method. The total loss function in this CycleGAN training is as follows.

1 $$L = L_{adv} + \lambda \times L_{cyc}$$

**Table 1:** Structures of the generator and discriminator, and gradient optimization conditions

Optimizer	Adam gradient descent method
Minibatch size	1
Initial learning rate	0.0002
Epochs	100
Encoder depth	Generator: 3, discriminator: 4
Convolution filter	Generator: 32, discriminator: 128
Residual block	6

Parameter λ controls the relative importance of the two types of losses; in this study, we set this value to 10. The adversarial loss is the loss function of the discriminator and generator that minimizes the difference between the expected and predicted output for the 4D-MSCT and 4D-CBCT images [19]. It is expressed as follows:

2 $$L_{adv} \left( {G_{Y} ,D_{Y} ,X,Y } \right) = {\mathbb{E}}_{y} \left[ {\log D_{Y} \left( y \right)} \right] + {\mathbb{E}}_{x} \left[ {{\text{log}}\left( {1 - D_{Y} \left( {G_{Y} \left( x \right)} \right)} \right)} \right]$$

where $G_{Y}$ attempts to generate volumes $G_{Y} \left( x \right)$ with input $x$ that are similar to the volumes in the target domain $Y$, whereas $D_{Y}$ aims to distinguish between $G_{Y} \left( x \right)$ and real samples $y$. The CycleGAN uses the cycle consistency loss to transform an image from domain $X$ to domain $Y$ using generator $Y$, and then transforms the result back to domain $X$ using generator $X$ to provide a good approximation of the original image. The cycle consistency loss is defined as the difference between the original and generated images that have been reconstructed back into the original domain [19]. Therefore, the cycle consistency loss can be expressed as

3 $$L_{cyc} \left( {G_{X} ,G_{Y} } \right) = {\mathbb{E}}_{x} \left[ \mid\mid{G_{X} \left( {G_{Y} \left( x \right)} \right) - x\mid\mid_{1} } \right] + {\mathbb{E}}_{y} \left[ \mid\mid{G_{Y} \left( {G_{X} \left( y \right)} \right) - y\mid\mid_{1} } \right]$$

Generators $G_{X}$ and $G_{Y}$ are trained to minimize the cycle consistency loss, whereas $D_{X}$ and $D_{Y}$ are adversarially trained to maximize the adversarial loss. Here, $X$ and $Y$ are images from the two domains (4D-CBCT and 4D-MSCT). As the training progresses, the reconstructed images more closely match the original images.

We conducted experiments on a personal computer equipped with two GPUs (Quadro RTX 5000, NVIDIA Corporation) and a CPU (Intel Xeon Sliver 4210R) with 96 GB memory. We implemented our algorithm using MATLAB 2021b (MathWorks Inc., Natick, MA, USA).

### Image quality evaluation

**Fig. 2:**
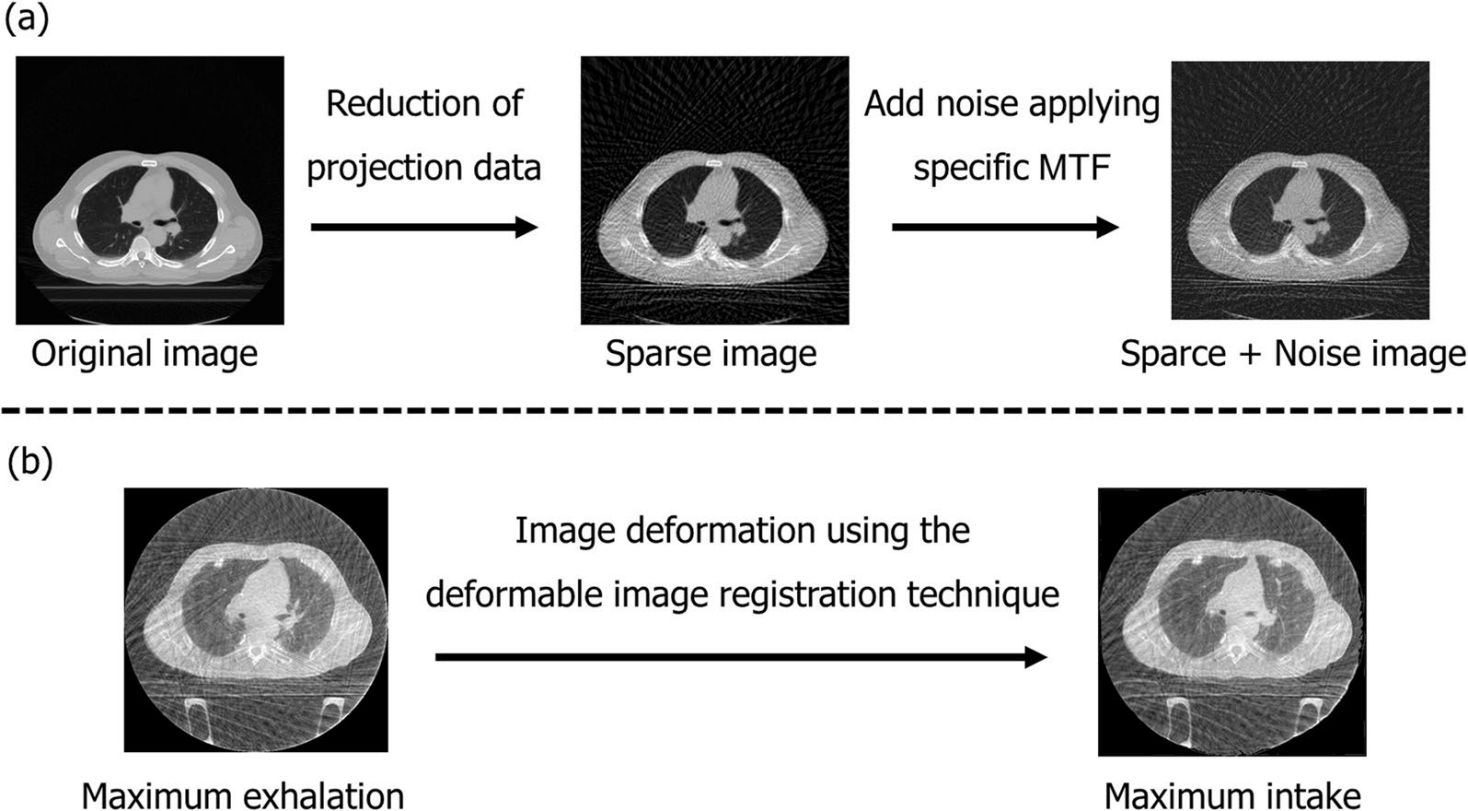
Creation of the virtual image quality deterioration image and deformation image. **a** The sparse projection data were acquired every 4° in a 360° direction. Image noise was added by applying convolution of 4D-CBCT-specific modulation transfer function. **b** The structural deformation was performed toward maximum exhalation to maximum intake images using the deformable image registration technique

To evaluate the accuracy of image improvement by the CycleGAN model, we added image noise and reduced the number of projection data for image reconstruction in the first-phase images of 4D-MSCT. The noise artifact was applying the specific modulation transfer function of the 4D-CBCT to double the dispersion of pixel value from the original image. Moreover, projection data was acquired every 4° from the reconstructed image in 4D-MSCT; then, image reconstruction was performed again with a total number of 90 projection data from 360 directions. Therefore, by creating the degraded 4D-MSCT image with the mathematical simulation, image quality improvement was verified compared with that of the original 4D-MSCT image. Figure 2a presents the overview of creating the virtual image quality deterioration dataset. Moreover, to evaluate the effects of structural deformation in the 4D-CBCT image with respiratory motion by the CycleGAN model, we created an image in which the maximum exhalation image was transformed toward the maximum intake image using the pixel-value-based deformable image registration technique [24]. Furthermore, these deformation and original maximum exhalation images were used for CycleGAN training. Figure 2b shows the overview of creating the deformation image. Then, we evaluated the image quality of the generated image and the original 4D-CBCT image compared to the maximum intake image. These image quality test and image deformation test were investigated in ten patients.

The synthesized images of 10 patients not included in the training data were generated using the trained CycleGAN model. The quality of the sCT images were quantitatively evaluated by comparing them with the original 4D-CBCT images. To evaluate the differences in CT number with respect to the 4D-MSCT images, we set regions of interest (ROIs) in lung, soft tissue, and bone regions and measured the ME and MAE as follows:

4 $$ME\left( {X,Y} \right) = \frac{1}{M \times N}\mathop \sum \limits_{i = 1}^{M} \mathop \sum \limits_{j = 1}^{N} (X\left( {i,j} \right) - Y\left( {i, j} \right))$$

5 $$MAE\left( {X,Y} \right) = \frac{1}{M \times N}\mathop \sum \limits_{i = 1}^{M} \mathop \sum \limits_{j = 1}^{N} \left| {X\left( {i, j} \right) - Y\left( {i, j} \right)} \right|$$

where $M$ and $N$ indicate the width and height in pixels within a ROI, $X\left( {i, j} \right)$ is the CT number of the ith and jth pixels in a sCT image or original 4D-CBCT image, and $Y\left( {i, j} \right)$ is the CT number of the ith and jth pixels in a 4D-MSCT image. The sizes of the ROIs were 35 × 35, 25 × 25, and 15 × 15 pixels in the lungs, soft tissues, and bones, respectively. Moreover, the differences in CT number were evaluated for the whole image. The ME and MAE differences between the sCT image and the original 4D-CBCT image were evaluated as statistically significant using the two-tailed *t* test. Moreover, the overall image quality was evaluated quantitatively using the SSIM and PSNR values in the sCT and original 4D-CBCT images based on the 4D-MSCT images [25, 26]. The SSIM of images $X$ and $Y$ was defined as follows:

6 $$SSIM\left( {X, Y} \right) = \frac{{\left( {2\mu_{X} \mu_{Y} + C_{1} } \right)\left( {2\sigma_{X} \sigma_{Y} + C_{2} } \right)}}{{\left( {\mu_{X}^{2} + \mu_{Y}^{2} + C_{1} } \right)\left( {\sigma_{X}^{2} + \sigma_{Y}^{2} + C_{2} } \right)}}$$

where $\mu_{X}$ and $\mu_{Y}$ are the average pixel values of the image pair ($X, Y$), $\sigma_{X}$ and $\sigma_{Y}$ are the variances, and the $C$ terms are regularization constants, where $C_{1}$ equals $\left( {0.01 \times 2000} \right)^{2}$, $C_{2}$ equals $\left( {0.03 \times 2000} \right)^{2}$, and 2000 is the dynamic range of the images. Furthermore, the PSNR was defined as follows:

7 $$\begin{aligned} & PSNR = 10\log_{10} \frac{{max\left| {X\left( {i,j} \right)} \right|^{2} }}{MSE} \\ & MSE = \frac{1}{M \times N}\mathop \sum \limits_{i = 1}^{M} \mathop \sum \limits_{j = 1}^{N} \left( {X\left( {i,j} \right) - Y\left( {i,j} \right)} \right)^{2} . \\ \end{aligned}$$

The PSNR is defined by the maximum value in an input image $X\left( {i,j} \right)$ divided by the mean squared error between image $X$(the sCT or original 4D-CBCT image) and image $Y$ (the 4D-MSCT image). In addition, $M$ and $N$ indicate the width and height of the images, respectively. To reduce the geometric mismatch between the sCT image and 4D-MSCT image, the sCT images were linearly registered to approach the corresponding pixel values of the 4D-MSCT image. In this process, to minimize the root-mean-square error of the corresponding pixel values between the two images, the sCT image was linearly shifted to the position of the MSCT image without image deformation processes. The differences in SSIM and PSNR of the sCT and original 4D-CBCT images were evaluated as statistically significant using the two-tailed *t* test.

### Evaluation of dose calculation accuracy

To determine the dosimetric accuracy, dose distributions and dose indexes based on the sCT image were evaluated. Dose distributions on the 4D-MSCT of volumetric modulation arc therapy (VMAT) plan with a dose of 48 Gy in four fractions were recalculated on the 4D-CBCT and sCT using the calculation algorithm from Acuros XB version 13.6 (Varian Medical Systems, Palo Alto, CA, USA). Additionally, the dose distributions were compared with the dose distribution calculated on the 4D-MSCT image using the 2D and 3D global gamma analysis with a 3% absolute dose and 2 mm dose to agreement criteria. Moreover, the dose-volume histogram parameters were evaluated in the clinical target volume (CTV), lungs, and spinal cord. For the CTV, D98%, D50%, and D2% were calculated; then, the lung volumes receiving a mean dose, 20 Gy and 5 Gy (mean, V20Gy and V5Gy) and the spinal cord dose with a volume of 2% (D2%) were investigated. These dose indexes were compared with those of the dose distribution based on the 4D-CBCT image. The contours of the CTV, lungs, and spinal cord were referenced on the 4D-MSCT image and transferred to the 4D-CBCT and sCT images using rigid image registration. Moreover, for all relative quantities, the value of the metric in the 4D-MSCT was used as the reference. Differences between the sCT and original 4D-CBCT images were considered statistically significant when *p* < 0.01 using the Wilcoxon signed-rank test.

## Results

### Image correction performance

**Fig. 3:**
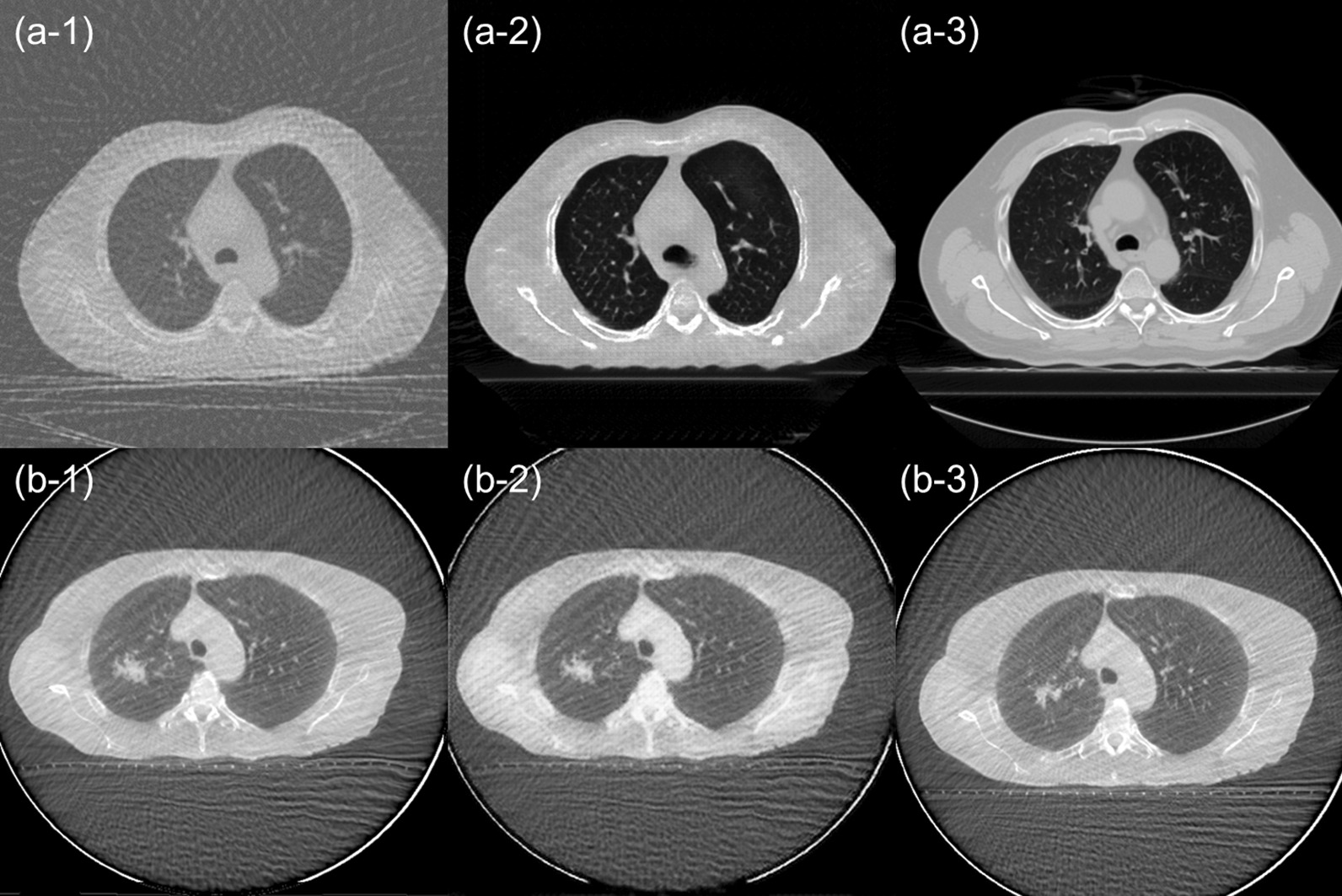
Results of the image quality test (**a**) and image deformation test (**b**). All images are shown with the same window width and levels. **a-1** Degraded 4D-MSCT image, **a-2** generated image, and **a-3** original 4D-MSCT image. **b-1** Original 4D-CBCT image in maximum exhalation, **b-2** generated image, and **b-3** 4D-CBCT image in maximum intake

**Fig. 4:**
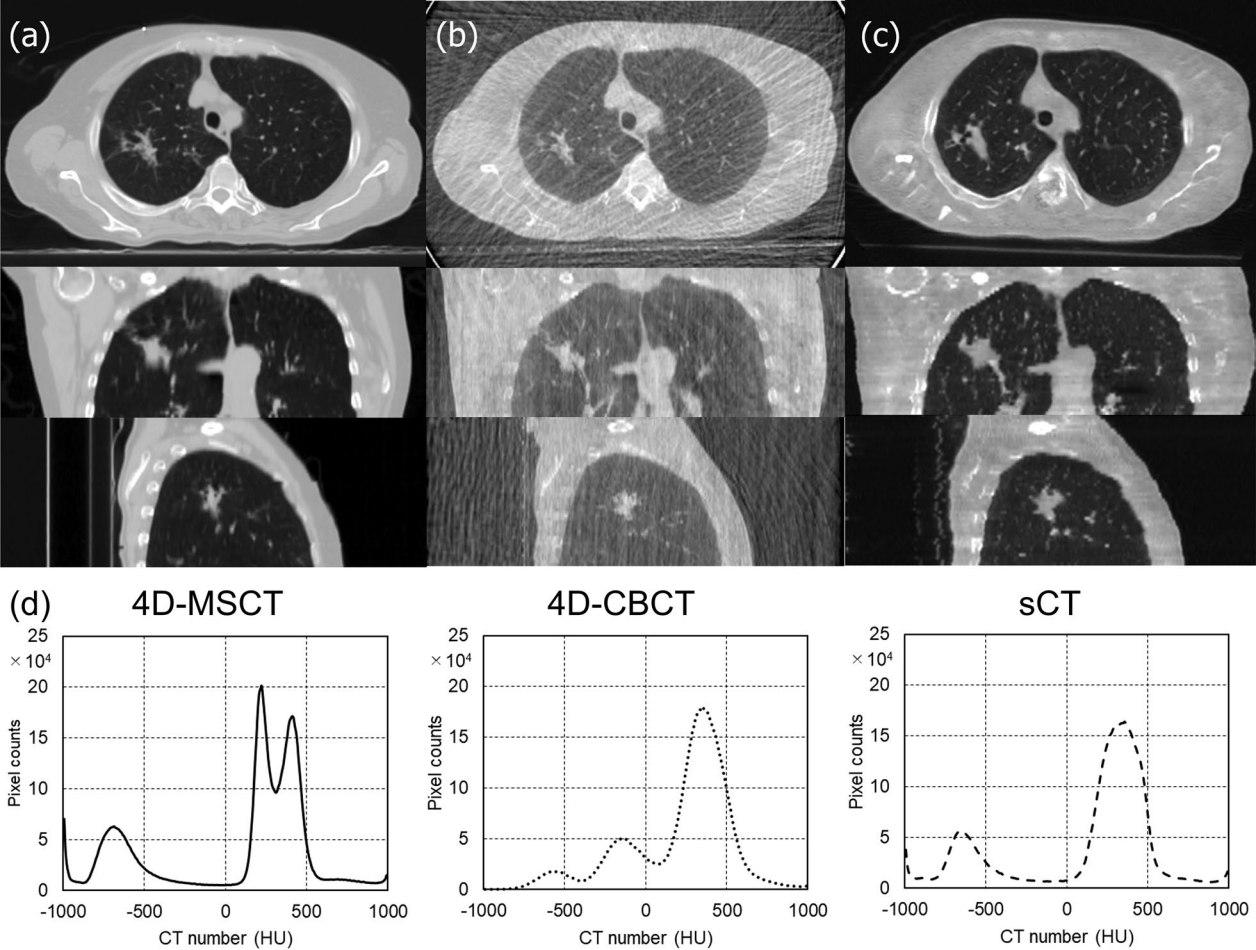
**a** 4D-MSCT, **b** 4D-CBCT, and **c** synthetic 4D-CBCT (sCT) images of the same patients in the corresponding axial, coronal, and sagittal directions. All images are shown with the same window width and levels. **d** Two-dimensional histograms of the axial direction in each image. The height of each histogram represents the count for the CT number

Figure 3 shows the patient results of image improvement and effects of image deformation by the CycleGAN training. Table 2 presents the results of SSIM and PSNR in these two experiments. In the result of image improvement test, SSIM and PSNR were significantly improved in 0.37–0.86 and 9.7–15.2 dB, respectively. Moreover, no significant difference in the synthetic image was shown in the result of image deformation test. Figures 2c, 3 and 4a show one of the patient results in the axial, coronal, and sagittal directions for the corresponding 4D-MSCT image, original 4D-CBCT image, and sCT image. In the 4D-CBCT images, there are significant artifacts, and overall image quality is poor. In contrast, we observed that the sCT image in each reformatted direction generated by our CycleGAN model effectively reduced the artifacts, especially in the lung region. Figure 4d shows the CT number distribution in two-dimensional histograms. The CT number distribution near the lung region (with under − 500 HU) in the sCT image was similar to that of the 4D-MSCT image. Table 3 lists the ME and MAE results in 10 patients as mean ± standard deviations for each site. In the results of the sCT image, the ME and MAE are significantly close to 0 HU in all regions. Table 4 presents the results of the comprehensive image evaluation using the SSIM and PSNR. These results are the mean value and standard deviations in 10 patients with respect to the 4D-MSCT images. The SSIM and PSNR results were significantly improved in the sCT image, increasing by approximately 51% and 18%, respectively.

**Table 2:** Results of the image quality test and image deformation test, by the quality index of structural similarity index (SSIM) and peak signal-to-noise ratio (PSNR)

Data set	SSIM	PSNR (dB)
Image quality test		
4D-MSCT	0.37 ± 0.04	9.7 ± 0.4
sCT	0.86 ± 0.04	15.2 ± 1.9
*p* value	< 0.01	< 0.01
Image deformation test		
4D-CBCT	0.43 ± 0.08	19.7 ± 1.7
sCT	0.42 ± 0.06	19.4 ± 1.5
*p* value	0.05	0.03

These values are reported as the mean ± standard deviation in 10 patients. The *p* value was calculated using a two-tailed *t* test that compared the results of the original and sCT images

**Table 3:** Mean error (ME) and mean absolute error (MAE) in the CT numbers of each site: lung, soft tissue, bone regions, and whole image

Site	Image	ME (HU)	MAE (HU)
Lung	4D-CBCT	595.3 ± 122.5	595.3 ± 122.5
	sCT	55.9 ± 46.7	64.3 ± 32.5
	*p* value	< 0.01	< 0.01
Soft tissue	4D-CBCT	378.1 ± 111.5	378.1 ± 111.5
	sCT	38.3 ± 92.8	72.9 ± 65.9
	*p* value	< 0.01	< 0.01
Bone	4D-CBCT	390.1 ± 93.0	390.1 ± 93.0
	sCT	− 7.5 ± 96.5	78.9 ± 49.5
	*p* value	< 0.01	< 0.01
Whole image	4D-CBCT	424.3 ± 88.4	424.3 ± 88.4
	sCT	111.1 ± 17.4	111.1 ± 17.4
	*p* value	< 0.01	< 0.01

These values were calculated with respect to the CT numbers in the 4D-MSCT images and are shown in terms of mean ± standard deviation for 10 patients in each site. The *p* value was obtained by a comparison of the results of the 4D-CBCT and sCT images using a two-tailed *t* test

**Table 4:** Results of the structural similarity index (SSIM) and peak signal-to-noise ratio (PSNR)

	SSIM	PSNR (dB)
4D-CBCT	0.49 ± 0.07	42.3 ± 1.4
sCT	0.73 ± 0.04	50.5 ± 1.4
*p* value	< 0.01	< 0.01

These values are reported as the mean ± standard deviation for 10 patients. The *p* value was calculated using a two-tailed *t* test comparing the results of the 4D-CBCT and sCT images

### Performance of dose calculation

**Fig. 5:**
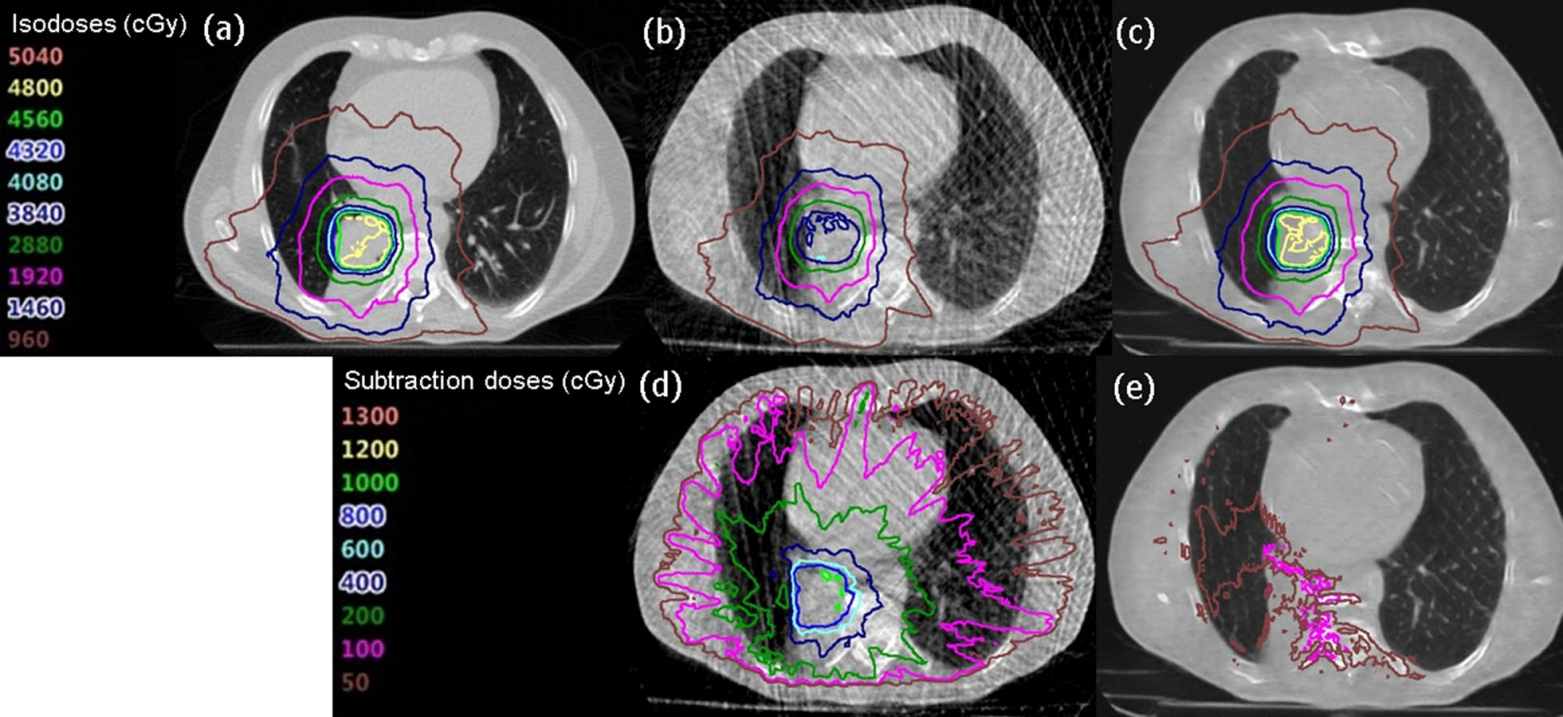
Dose distributions in a volumetric modulation arc therapy (VMAT) plan based on **a** 4D-MSCT, **b** 4D-CBCT and **c** sCT images. Moreover, the dose difference image from the dose distribution of 4D-MSCT image, **d** 4D-MSCT minus 4D-CBCT, **e** 4D-MSCT minus sCT

**Fig. 6:**
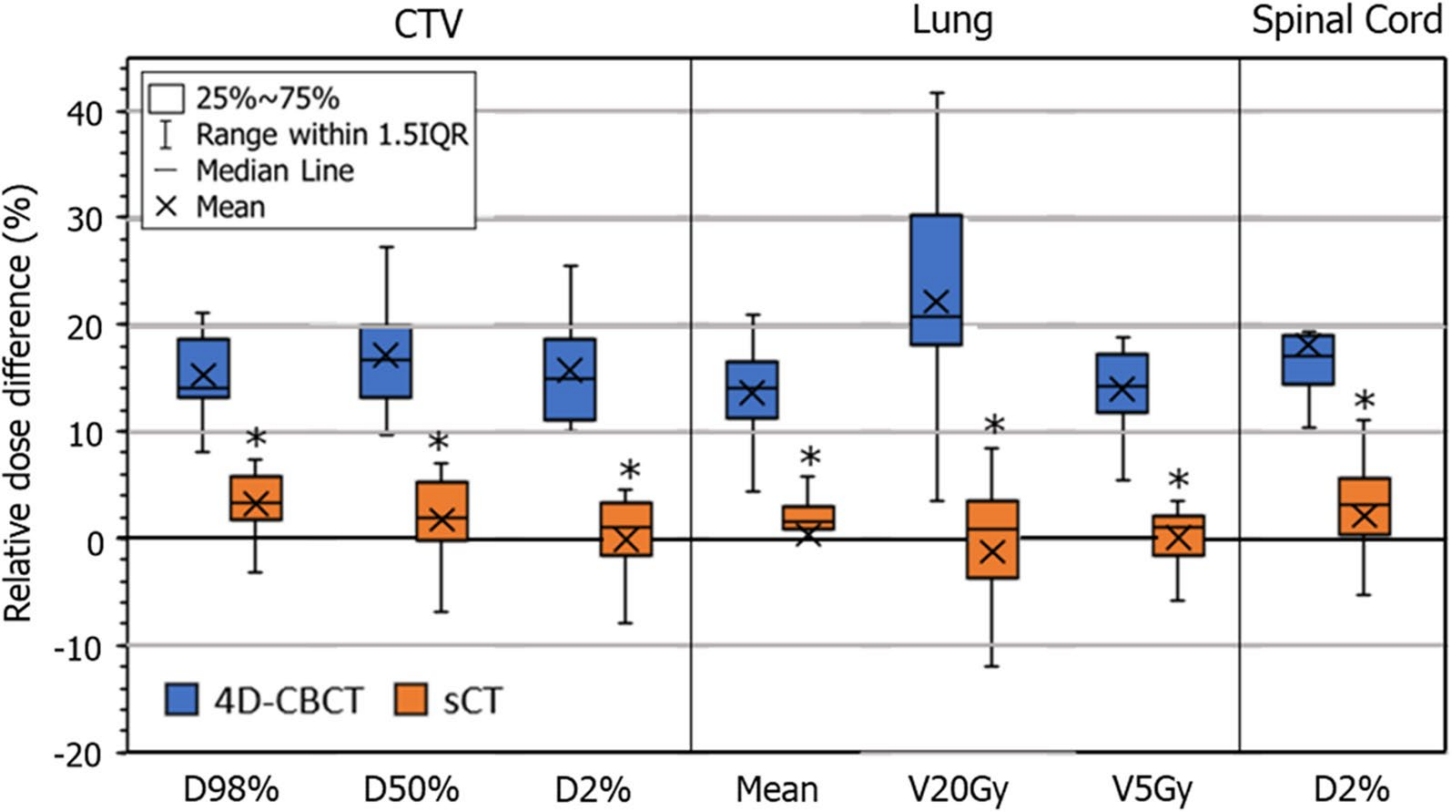
Relative differences in each organ dose index obtained using the 4D-MSCT, 4D-CBCT, and sCT images in VMAT-SBRT planning. All doses correspond to the reference dose on the 4D-MSCT image. * indicates significance at *p* < 0.01

Figure 5 shows the results of one patient for the calculated dose distribution in VMAT-SBRT for the corresponding 4D-MSCT image, original 4D-CBCT image, and sCT image. Furthermore, the dose difference images from the dose distribution of 4D-MSCT image are shown. The monitor unit, movement of the multi-leaf collimator, and gantry rotation were set to the same conditions. The dose distribution of over 4000 cGy isodose curves obtained using the 4D-CBCT image differs from those obtained using the 4D-MSCT image. In contrast, the dose distribution obtained using the sCT image agreed well with it. Table 4 presents the results of the gamma analysis in each CT based dose distribution, comparing them with those obtained using the 4D-MSCT image. The pass rates in the 2D and 3D analysis were significantly improved when the sCT image was used. The pass rate reached over 90% in the recalculation doses using the sCT image. Figure 6 shows the differences in each organ dose index for the dose distributions obtained using the 4D-MSCT, 4D-CBCT, and sCT images in VMAT-SBRT. The dose differences obtained using 4D-CBCT were mostly over 15% in all regions. Moreover, the deviations in V20Gy in the lung region were extremely large, that is, from 5% to over 40%. In contrast, dose indexes of the sCT image agreed with those of the 4D-MSCT image within 5% in all regions. Moreover, deviations in V20Gy in the lung region decreased and were within approximately 10%.

## Discussion

To realize the ART based on the synthetic 4D-CBCT image generated by the CycleGAN model, quantitative evaluation of image quality and deformation effect has to be clarified by controlled experiments. Therefore, quality degradation and structural deformation images were mathematically created and compared with original ground truth images. In the result of Table 2, sCT image was significantly improved, close to the image quality of the original image by the CycleGAN model. Conversely, since the similarity of the sCT was not significantly different from the result of the input initial phase image, the structure deformation under natural respiration was small using the CycleGAN model. We generated sCT images using a CycleGAN with unpaired 4D-CBCT and 4D-MSCT images. To perform accurate image-guided radiotherapy and use 4D-CBCT-based ART, the HU values must be restored to achieve sufficient dose calculation accuracy. The 4D-CBCT projection data were collected at 5.5 frames per second in approximately 3–4 min, spanning a 360° gantry rotation, and divided into 10 phases. Therefore, in the results shown in Fig. 4, significant image degradation in the original 4D-CBCT was caused by the number of projection data, which was much lower than that of the 4D-MSCT. In contrast, the sCT image generated by the CycleGAN had substantially fewer streak artifacts and less image noise, bringing it closer to an ideal 4D-MSCT image in quality. In the two-dimensional histogram (Fig. 4d), the CT number distribution in the sCT image is close to that of the 4D-MSCT image. In particular, our results showed good composite images in the lung region. Moreover, as the results of CT number deviation in Table 3 reveal, the pixel values of the sCT images were close to those of the 4D-MSCT images, demonstrating a significant difference with respect to the 4D-CBCT images in all regions. The ME and MAE values of the lung regions were 55.9 and 64.3 HU, respectively, i.e., the MAE was large because of the biased deviation in pixel values due to the influence of respiratory motion in the 4D-CBCT images, as these images were acquired in different periods of the scanning process. However, the ME and MAE results in the lung, soft tissue and bone regions were similar to those of previous studies [20, 21, 23]. In the results of Table 4, the SSIM and PSNR values of the sCT images were significantly higher than those of the original 4D-CBCT images. In our study, because restoration in all regions was remarkably good, the evaluation of the whole image was greatly improved, demonstrating that the entire sCT image could resemble the 4D-MSCT image. This image quality improvement was achieved in the upper, middle, and lower parts of the thoracic region because the training data included all lung areas. Moreover, the artifacts of the 4D-CBCT images occurred under approximately the same conditions for all respiratory phase images. Hence, the image quality in single-phase data could be connected with the accurate restoration of other phase images in the same manner. However, the addition of different respiratory phase images in model training may provide further robustness in the image quality conversion of the 4D images. Improving the visibility of 4D-CBCT images enhances the tumor and surrounding organ visibility in radiotherapy, increasing the accuracy of target localization. In previous studies, a CycleGAN improved the quality of CBCT images, and our results are in agreement with these results [23, 27]. Furthermore, HU value restoration may enable accurate contouring warping in daily 4D-CBCT images. Because warping is essential for realizing online ART, the generation of synthetic CT images using CycleGAN enhances the feasibility of ART using 4D-CBCT.

In the results of dose distribution using sCT image shown in Fig. 5, each isodose curve was close to those of the 4D-MSCT image, and the dose difference image indicated that the dose error was decreased, revealing that the dose distribution obtained using sCT agrees with that obtained using 4D-MSCT. Moreover, the average pass rates of gamma analysis exceeded 90% in the sCT images, and significant improvements were found in the results of Table 5. The 2D-Cycle GAN model could cause dosimetric errors in the direction of the body axis. However, the pass rate of 2D gamma in sagittal and coronal directions did not decrease, and dose distribution in the sCT image was quite close to that of the 4D-MSCT image. Accurate dose calculation relies on accurate HU values and conversion to electron density. The CT number restoration of the 4D-CBCT to 4D-MSCT transformation improved dose calculation accuracy in radiotherapy planning. In the result of dose-volume histogram analysis, the differences in each organ dose index were close to 0% in the sCT image-based results. The V20Gy of the lung region had a large deviation in the 4D-CBCT image because, in the VMAT plan with a rotation of 360°, changes in the CT number in the lung region increased the dosimetric error. In contrast, the restoration of the CT number in the sCT image reduced the dosimetry indexes in all organ regions. Therefore, sCT images should have sufficient image quality for accurate dose calculation in a lung SBRT plan. In a previous study, Gao et al. proposed a synthetic CBCT using a CycleGAN, and revealed that dose distribution could be close to the original MSCT-based plan with a gamma pass rate of over 90% [28]. In our results, the difference in dose index was within approximately 5%, which is in agreement with the results of previous studies [21, 29].

**Table 5:** Mean pass rates of the 2D and 3D gamma in 10 patients with respect to the dose distribution obtained using the 4D-MSCT images

	Pass rate (%)			
	2D gamma (3%/2 mm)			3D gamma (3%/2 mm)
	AX	SAG	COR	
4D-CBCT	74.7 ± 10.7	75.2 ± 14.1	83.6 ± 6.1	76.5 ± 10.8
sCT	95.7 ± 4.7	94.8 ± 5.3	92.5 ± 5.5	96.2 ± 4.2
*p* value	< 0.01	< 0.01	< 0.01	< 0.01

The 2D gamma was evaluated in three dose distribution directions. The *p* value was calculated using a two-tailed *t* test comparing the results of the 4D-CBCT and sCT images

The 4D-CBCT image cannot be used to train a model using paired supervised data because that approach depends on the reproducibility of respiratory movements. A CycleGAN can be used with non-paired training data and is very suitable for this synthetic image generation task.

Therefore, the sCT images generated by our CycleGAN could be used to improve the accuracy of image-guided radiotherapy and achieve sufficient dose calculation accuracy for ART. However, the limitation of our evaluation of the sCT image is that the accuracy was based on the MSCT image in which the anatomical structure was slightly displaced due to the respiratory movement. Moreover, complete synthesis of the bone region could not be realized because the small amount of bone area in the whole image may be the cause of deterioration of learning accuracy. However, dose calculation accuracy affected by this bone defect region was not critical degradation. In previous studies, 3D data have been used for training the CycleGAN, e.g., by dividing it into voxel units, showing that CycleGAN can enhance the accuracy of the 3D structural information [29–31]. However, training a model with image feature details using a limited number of data is considered difficult [30]. Moreover, the input of 3D volume data consumes a considerable amount of GPU video memory when running the network. Therefore, we employed a 2D CycleGAN, which limits the amount of training data by targeting 2D images. To realize 4D-CBCT-based ART, streaking and motion artifacts must be reduced to reveal material boundaries and create accurate treatment plans based on 4D-CBCT images. Moreover, an adaptive treatment process within clinical tolerances is a necessary step toward the clinical implementation of ART in conventional practice. Therefore, the effects of warping accuracy with organ contouring and dose distribution must be further improved in the sCT images. Thus, verification of the contour propagation accuracy associated with image quality improvement and prediction of dose distribution using new deep-learning techniques remain as future tasks.

## Conclusions

Image artifacts were corrected and image quality was improved in 4D-CBCT images using a CycleGAN, and bringing them closer to the image quality of 4D-MSCT images. Thereby, dose calculation accuracy using sCT images was significantly improved, indicating that 4D-CBCT-based ART in lung cancer is feasible.

## Data Availability

Research data are stored in an institutional repository and will be shared upon request to the corresponding author.

## Abbreviations

SBRT: Stereotactic body radiotherapy
CBCT: Cone-beam computed tomography
4D-CBCT: Four-dimensional cone-beam computed tomography
CT: Computed tomography
ART: Adaptive radiation therapy
HU: Hounsfield unit
TV: Total variation
4D-MSCT: Four-dimensional multislice computed tomography
sCT: Synthetic four-dimensional multislice computed tomography
CycleGAN: Cycle generative adversarial network
ME: Mean error
MAE: Mean absolute error
PSNR: Peak signal-to-noise ratio
SSIM: Structural similarity index
ROIs: Regions of interest
VMAT: Volumetric modulation arc therapy
CTV: Clinical target volume
